# Conjunctival Necrosis and Scleritis Following Subtenon Triamcinolone Acetonide Injection

**DOI:** 10.5812/ircmj.5223

**Published:** 2013-07-05

**Authors:** Alireza Eslampour, Mojtaba Abrishami, Somaye Tafaghodi

**Affiliations:** 1 Cornea Research Center, School of Medicine, Mashhad University of Medical Sciences, Mashhad, IR Iran

**Keywords:** Conjunctival Necrosis, Triamcinolone acetonate, Subtenon Injection, Complications, Corticosteroids

## Abstract

The present study aims to report a case of conjunctival necrosis and scleritis due to a subtenon injection of triamcinolone acetonate. A 15-year-old boy received a subtenon injection of triamcinolone acetonate after a pars plana vitrectomy due to an intraocular foreign body. Seven days later, conjunctival necrosis and scleritis appeared at the site of injection. No improvement was seen after seven days of conservative treatment, and necrotic tissue debridement was performed. Within one week the conjunctiva cleared. Conjunctival necrosis and scleritis are rare complications of periocular corticosteroid injections, but an early diagnosis can be very valuable. Improper dosage and injection site of corticosteroids with insufficient prophylactic antibiotics are predisposing factors. If conservative treatment is not sufficient, debridement should be considered as a potential critical treatment option.

## 1. Introduction

The more widespread periocular corticosteroids are used in clinical treatment, the more we face clinical complications as a result of usage. Periocular injections of depot corticosteroids are often used to treat chronic ocular inflammatory diseases (1), and increased intraocular pressure and cataract formations are well-recognized complications of periocular corticosteroid therapy (2, 3). Recently, a few cases of conjunctival necrosis were reported after subtenon corticosteroid injection. Here we report a case of conjunctival necrosis and scleritis due to a subtenon injection of triamcinolone acetonate (TA) and the subsequent medical and surgical management.

## 2. Case Presentation

**Figure 1.**
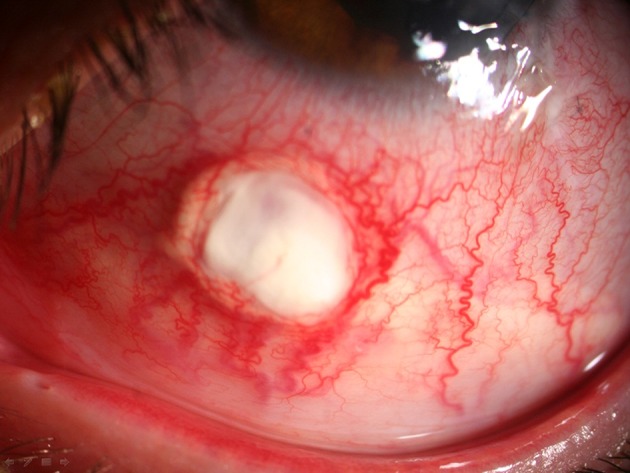
Necrotizing Sclerititis With Conjunctival Necrosis

**Figure 2.**
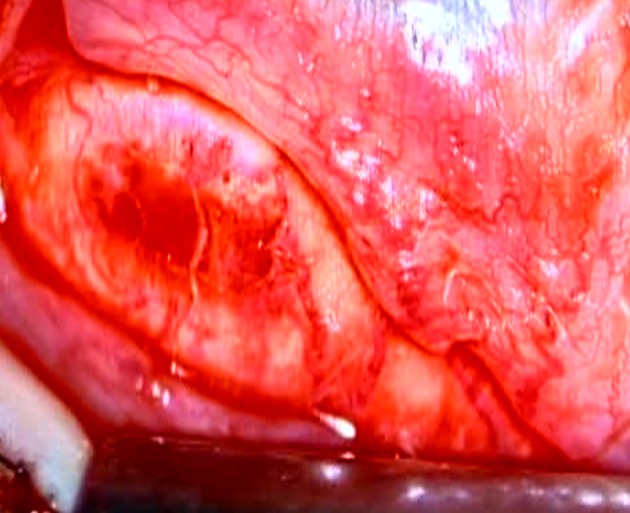
Sclera Beneath the Epithelial Defect was Inflamed by Engorged Vessels and Mild Thinning

**Figure 3.**
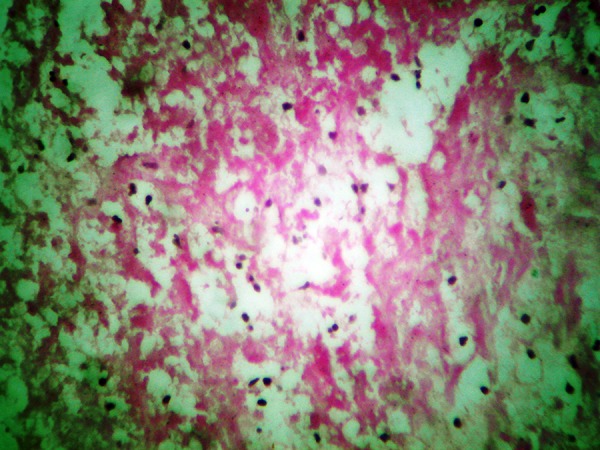
Pathology Report was Connective Tissue With Necrosis and PMN Infiltration

A 15-year-old boy was referred to the emergency department with an open globe injury and metal intraocular foreign body. Corneal primary repair was performed on the first day. Ten days later, a lensectomy and pars plana vitrectomy was successfully performed and the foreign body was removed. At the end of the operation, 0.5 cc of TA suspension (40 mg/ml; TriamHexal,Hexal, Germany) was injected by 27G needle under the inferior bulbar tenon. Some of the TA dispersed subconjunctivally, as noted by the treating surgeon. The patient was discharged with 75 mg oral prednisolone (Prednisolone Fort 50, Hakim Co, Tehran, Iran) daily, topical betamethasone (Betasonate, Sinadaru, Tehran, Iran) drops every four hours, and topical chloramphenicol (Cholobiotic, Sinadaru, Tehran, Iran) drops every eight hours. Seven days later, the patient was referred to the emergency department with severe ocular pain. Upon examination, the conjunctiva and sclera seemed to be inflamed and necrotic, and the surrounding vessels were markedly congested with conjunctival epithelial defects (5 × 4 mm) (Figure 1). All previous medications were terminated and a sample of the conjunctival ulcer was sent for smear evaluation. The results showed 8-10 white blood cells in each field. Since the source was suspected to be infectious, fortified antibiotic drops of vancomycin (25 mg/ml) and ceftazidim (50 mg/ml) was ordered every hour. Moreover, treatment with preservative-free artificial tears (Artelac Advanced, Chauvin-Bausch and Lomb, Montpellier, France) every three hours was initiated. After two days, the epithelial defect had decreased in size (0.5 mm circumference) and the patient’s pain had substantially declined. A culture of the conjunctival specimen indicated the presence of Staphylococcus saprophyticus. After five days, the epithelial defect had decreased in size (3 × 2 mm), but the necrotic tissue failed to clear, and an excision of the necrotic tissue was proposed for the patient. Necrotic materials, scant remnants of the TA deposits, and inflamed tenon tissue around them were excised. The sclera beneath the epithelial defect site was inflamed by engorged vessels and mild thinning (Figure 2). The normal conjunctiva was sutured with nylon 10-0 and fortified drops were discontinued. The revised treatment included topical ciprofloxacin drops (Ciplex 1%, Sinadaru, Tehran, Iran), a gel form of artificial tears (Liposic, Chauvin-Bausch and Lomb, Montpellier, France) every six hours, and topical fluorometholone (1 mg/mL; FML, Allergan, Australia) every 12 hours. The pathology report documented connective tissue with necrosis and PMN infiltration, which confirmed the diagnosis (Figure 3). After one week, the conjunctival sutures were removed and the conjunctiva healed with minimal scarring.

## 3. Conclusions

Periocular corticosteroid injections are often used after intraocular surgery and various inflammatory ocular diseases (1). This drug delivery system has become more popular in recent years due to prolonged, highly concentrated drug activity with minimal systemic side effects. The main potential side effects include ocular hypertension and cataract formation (2, 3). To our knowledge, only 10 cases of conjunctival necrosis have been reported after corticosteroid injection, including six patients treated with methylprednisolone, four patients treated with TA (3-8), and one patient treated with betamethasone (8). The patient described in this case report was the 5th case of conjunctival necrosis after TA injection and the second case that was accompanied with scleritis. Most of the reported cases have been occurred after subconjunctival injections or, similar to this case, after subtenon injection with dispersion to the subconjunctival space. We suggest that an accurate drug dosage, and more importantly, deposition of the steroid in the subtenon instead of the subconjunctival space can most likely prevent conjunctival toxicity. In addition, we hypothesize that an improper site of administration, such as the inferior bulbar conjunctiva within the interpalpebral fissure, may be another predisposing factor for conjunctival necrosis due to greater exposure and proximity to the lower lid margin. In this case, the depot TA injection together with a high dose of oral prednisolone and betamethasone drops in addition to low dose antibiotic coverage exposed this patient to infections and necrosis. Although the culture was reported as Staphylococcus saprophyticus, which is usually present in the normal flora, the marked response to fortified antibiotic drops as seen by the substantial decrease in the pain and discomfort of the patient supports this infectious etiology. Since local high-dose corticosteroids are released slowly, we believe that there is no need to order high-dose oral steroids and frequent topical steroids in patients receiving a depot subtenon steroid injection. In addition, a prep and drape prior to injection and broad spectrum antibiotic coverage after injection are both beneficial for infection prophylaxis. Debridement as a major treatment approach has previously been used in three cases, including one case of subconjunctival betamethasone treatment, one case of an intravitreal injection with egression to the subconjunctival space, and the case reported here (8, 9). We decided to excise the necrotic tissue and remove the TA deposit after the poor response to conservative therapy. Importantly, a poor response to medication, accompanied by scleritis and non-healing necrotic tissue with a large depot of long-acting corticosteroids may increase the need for debridement.
